# Chemical Compositions and Chromatic Mechanism of High-Temperature Iron-Series Glazed Wares from the Guangyuan Kiln in Sichuan Province, Southwest China During the Song Dynasty

**DOI:** 10.3390/ma17246221

**Published:** 2024-12-19

**Authors:** Lin Wu, Yourongtian Nie, Jinwei Li, Junming Wu, Wei Shi, Yanfang Wu, Yueguang Jiang

**Affiliations:** 1 Research Center of Ancient Ceramic, Jingdezhen Ceramic University, Jingdezhen 333001, China; 2 Jiangxi Ceramic Heritage Conservation and Imperial Kiln Research Collaborative Innovation Center, Jingdezhen 333001, China; 3 School of Archaeology and Museology, Jingdezhen Ceramic University, Jingdezhen 333001, China; 4 National Engineering Research Center for Domestic & Building Ceramics, Jingdezhen Ceramic University, Jingdezhen 333001, China; 5 Key Laboratory of Traditional Heated-Form Craft Technology and Digital Design, Ministry of Culture and Tourism, China Academy of Art, Hangzhou 310024, China; 6 Jingdezhen Ceramic Research Institute, Jingdezhen 333001, China

**Keywords:** Guangyuan kiln, iron-series glazed ware, Fe_2_O_3_ content, ε-Fe_2_O_3_ crystals, chromatic mechanism

## Abstract

The Guangyuan kiln, located in the Sichuan Province, Southwest China during the Song Dynasty (960–1279 A.D.), is renowned for its high-temperature iron-series glazed wares, including pure black glazed ware, hare’s fur glazed ware, glossy brown glazed ware, and matte brown glazed ware. To elucidate the raw materials, processing techniques, and coloration mechanisms of these wares, multiple analytical experiments were employed to investigate chemical composition, microstructure, and the phase of Fe-bearing minerals. We found that glossy brown glazed ware has the highest Fe_2_O_3_ content in the glaze (7.67 wt% on average), while pure black glazed ware exhibits the lowest (4.84 wt% on average). Higher Fe_2_O_3_ content leads to more iron for Fe-bearing mineral crystallization and larger ε-Fe_2_O_3_ precipitation. Based on microscopic observations, pure black glazed ware has numerous 100–250 nm crystalline grains, while hare’s fur glaze ware features dendritic crystal flowers (200–400 nm), which exhibited liquid-liquid phase separation within the glaze, suggesting localized phase separation inducing iron oxide crystallization. Glossy brown glazed ware contains well-developed ε-Fe_2_O_3_ crystals (25 µm), and matte brown glazed ware, with the highest CaO and total flux, has acicular anorthite crystals alongside ε-Fe_2_O_3_ crystals. In summary, the decorative effect of four different types of iron-series glazed wares is determined by their chemical composition, phase composition, and microscopic structure. The findings offer valuable insights for the study of ancient iron-glazed ware.

## 1. Introduction

High-temperature iron-series glazed ware refers to ceramic glaze adorned with brown to black hues achieved by incorporating a substantial quantity of iron oxide and firing at high temperatures (>1200 °C). In the history of Chinese ceramics, the Song Dynasty (960–1279 AD) represents a golden age for the development of high-temperature iron-series glazed ware. Through the skillful manipulation of various firing conditions, ancient artisans created a diverse array of unique glaze types, such as hare’s fur glazed ware, partridge feather mottled glazed ware, oil spot glazed ware, and tortoiseshell glazed ware. In addition to the renowned Jian kilns of Fujian Province, Southeast China, and Jizhou kilns of Jiangxi Province, South Central China, numerous kilns produced such wares [1]. Among these, the Guangyuan kiln, a representative folk kiln in the Sichuan Province, Southwest China, yielded distinctive high-temperature iron glazed ware, establishing itself as a key producer of this type of ceramic during the Song Dynasty in the region [2].

In recent years, the scientific analysis of ancient Chinese high-temperature iron-series glazed wares represents a significant focus in the field of ancient ceramics. Scholars have conducted in-depth studies on black glazed ware and brown glazed ware unearthed from different periods, and the existing studies have also confirmed that the appearance color of the high-temperature iron glaze is closely related to the concertation and phase state of iron [3,4,5,6,7,8]. There are various iron oxide crystals present in iron glazes, mainly α-Fe_2_O_3_, β-Fe_2_O_3_, ε-Fe_2_O_3_, and Fe_3_O_4_. The different phase types within the glaze determine the coloration and artistic effects of the ware. However, the chromatic variations and the underlying formation mechanisms of high-temperature iron-series glazed ware remain comparatively understudied. Moreover, the majority of the existing studies focus on samples excavated from kilns in southeastern and south-central China, with comparatively limited research dedicated to products from the southwestern region. The Guangyuan kiln, notably producing both black and brown glazed ware from southwestern China, presents an ideal case study for investigating these issues.

The Guangyuan kiln primarily produced everyday utensils for the common people. Judging from the archaeological findings, the characteristics of the Guangyuan kiln’s products are very similar to those of the “Jian kiln”, especially in terms of the shapes and glazes of the vessels. It also shared common features with other contemporary southern folk kilns such as the Jizhou kiln in Jiangxi Province, the Wuyi kiln in Zhejiang Province, and the Bachun kiln in Henan Province [9]. Current research concerning the high-temperature iron-series glazed ware of the Guangyuan kiln is primarily limited to archaeological surveys, vessel typology, and decorative motifs [10,11,12,13,14]. Comprehensive scientific analyses of the glaze’s chemical composition and microstructure of the high-temperature iron-series glazed ware from the Guangyuan kiln remain relatively scarce. 

At present, researchers use a variety of analytical techniques to detect and analyze ceramics. These include X-ray fluorescence spectroscopy (XRF), scanning electron microscopy-energy dispersive X-ray spectroscopy (SEM-EDS), Raman spectroscopy (Raman), inductively coupled plasma mass spectrometry (ICP-MS), laser-induced breakdown spectroscopy (LIBS), and X-ray diffraction (XRD). These methods can effectively demonstrate the production process, raw material origin, coloring mechanism, and the relationship between different kilns [15,16,17,18,19,20,21]. Considering that ICP-MS and LIBS are used to obtain the trace element content of samples, the main purpose is to trace the origin information of ancient ceramics, and for the samples unearthed at the kiln site, the origin information is clear. Therefore, this study employed energy-dispersive XRF (EDXRF), ultra-depth-of-field microscopy, SEM-EDS, Raman spectroscopy, and XRD to characterize the body and glaze chemical composition, microstructure, and crystalline phases present in high-temperature iron-series glazed wares produced in the Guangyuan kiln. The aim is to elucidate the compositional characteristics, structural features, and color formation mechanisms of these wares, thereby revealing the compositional and structural variations in ancient Chinese high-temperature iron-series glazed wares and the chemical states of iron within the glaze matrix.

## 2. Experiment

### 2.1. Sample Description

**Figure 1:**
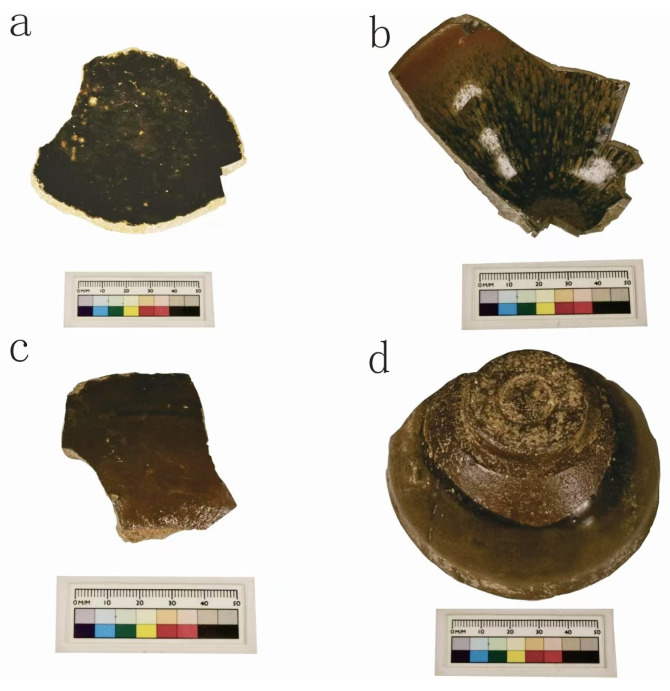
Photographs of selected Guangyuan kiln samples. (**a**). Pure black glazed ware GY-7; (**b**). Hare’s fur glazed ware GY-10; (**c**). Glossy brown glazed ware GY-14; (**d**). Matte brown glazed ware GY-22.

This study examined a representative selection of 22 high-temperature iron-series glazed wares from the Guangyuan kiln. Based on glaze color, the samples were categorized into black and brown glazes. Black glazes were further classified as pure black and hare’s fur, while brown glazes were divided into matte and glossy types. Selected samples are illustrated in Figure 1, and their macroscopic characteristics are detailed in Table 1.

### 2.2. Analytical Methods

In this study, we employed EDXRF, ultra-depth-of-field microscopy, SEM-EDS, Raman, and XRD to analyze the composition and microstructure of the bodies and glazes of the samples. 

The EAGLE-III EDXRF spectrometer produced by EDAX was used to quantitatively analyze the chemical composition of the embryo and glaze of the samples from the Guangyuan kiln. The instrument is equipped with a rhodium target with a side window, Si(Li) detector, test beam spot diameter of 300 μm, vacuum light path, X-ray tube voltage of 50 kV, tube current of 200 mA, and data acquisition time of 200 s. In order to ensure the accuracy of the test, the subject uses a special series of standard reference materials for non-destructive testing of ancient ceramics developed by the Shanghai Institute of Ceramics, Chinese Academy of Sciences, and establishes the standard curves of each element with the software Delta-I. Before the test, the sample was cut into small pieces. The test part was the surface of the glaze and body.

The microstructure observations were initially employed by a VHX-600 ultra-depth-of-field optical microscope (Keyence, Japan). Then, we conducted deep observations of important areas using a Prisma tungsten filament SEM. First, the sample to be observed was cut into chunks, and the cut sample was immersed in 5% HF and corroded for 120 s. After removal, the sample was cleaned with an ultrasonic cleaning machine for 20 min, and then the sample was placed in an electric blast oven and dried for 5 h at a constant temperature of 60 °C. After that, the dried sample is taken out and a thin layer of Pt is sprayed on the glaze surface of the sample to be observed to increase the conductivity. It is equipped with Thermo Feld Ultradry spectrum for the composition analysis of the crystal on the surface of the sample glaze, with conditions: acceleration voltage 10 KV, working distance 10 mm, and data acquisition time of approximately 30 s.

Raman spectrometer produced by Renishaw in the UK was used to characterize the crystal phase precipitated from the surface of the sample glaze layer. Test conditions were as follows: laser wavelength of 532 nm, spot size of 1 μm, laser power of 0.2 MW, signal acquisition of 60 s, and spectral range of 50–1850 cm^−1^. For the samples with weak Raman characteristic peaks of the glaze surface crystals, a secondary test was conducted. The laser power was increased to 10 MW, and the signal acquisition was for 20 s. Other test conditions remained unchanged. The samples were also cut into blocks and placed in 5% HF for corrosion for 120 s, followed by ultrasonic cleaning for 20 min; and then dried at 60 °C for 5 h.

D8 Advance A25 X-ray diffractometer produced by Bruker in Karlsruhe, Germany was used to qualitatively analyze the crystal phase on the glaze surface of the sample. Test conditions: Cu target Kα (λ = 0.1518 nm), test angle range of 5°–80°, scanning speed of 0.02°/step, 0.2 s/step. The sample was cut and tested directly on the enamel surface of the sample.

## 3. Results and Discussion

### 3.1. Body and Glaze Chemical Composition

Table S1 presents the chemical composition of the samples’ bodies. As evident from the table, the body composition exhibits considerable variation. Al_2_O_3_ content ranges from 16.33 wt% to 37.44 wt%, while SiO_2_ content fluctuates between 56.21 wt% and 74.93 wt%. K_2_O and Na_2_O content varies from 1.54 wt% to 4.91 wt%, and MgO content lies between 0.8 wt% and 2.47 wt%. TiO_2_ and Fe_2_O_3_ content also demonstrates significant variability, ranging from 0.49 wt% to 2.08 wt% and 1.05 wt% to 6.29 wt%, respectively. The bodies are characterized by generally high Al_2_O_3_ content and appreciable quantities of both MgO and TiO_2_, suggesting the utilization of sedimentary clay minerals in their production [22]. Furthermore, the bodies contain a notable amount of iron, averaging 3.59 wt%, which aligns with the prevalence of iron-rich clay resources in the region [23]. This supports the hypothesis that ancient potters in the Guangyuan kiln utilized local raw materials characterized by a high iron content for their bodies. However, the Fe_2_O_3_ content in the bodies of the samples is significantly lower than that found in the wares of the Jian kiln, which averages 7.34 wt% [24]. Consequently, the sample bodies are predominantly gray, contrasting with the characteristic black bodies of the Jian kiln. The iron content across different porcelain body types does not exhibit a discernible trend, suggesting a degree of uniformity in the source of raw materials for the ceramic bodies. However, the raw materials themselves exhibit significant heterogeneity, compounded by potentially less refined processing techniques, which likely account for the observed heterogeneity in the chemical composition of the ceramic bodies.

To investigate potential compositional variations in the bodies of different glazed wares, we employed hypothesis testing to assess the consistency of major elements in both black and brown glazed samples. The results, detailed in Table 2, reveal no statistically significant differences (*p*-values > 0.05) in the concentrations of SiO_2_, Al_2_O_3_, Fe_2_O_3_, K_2_O, CaO, Na_2_O, MgO, and TiO_2_ between the two types [25]. Consequently, we infer that the porcelain bodies of black and brown glazed wares exhibit no discernible compositional divergence, suggesting the probable utilization of identical raw materials in their fabrication.

The chemical compositions of the glazes of the samples are presented in Table S2. Al_2_O_3_ content in the glazes ranges from 11.09 wt% to 15.46 wt%, while SiO_2_ content varies between 62.70 wt% and 70.74 wt%. Fluxing agents K_2_O and Na_2_O are present in concentrations between 2.50 wt% and 5.43 wt%, with CaO content falling between 2.7 wt% and 10.36 wt%. Fe_2_O_3_ content ranges from 3.66 wt% to 8.13 wt%, and TiO_2_ content lies between 0.37 wt% and 0.7 wt%. The results indicate that both Fe_2_O_3_ and TiO_2_ concentrations are elevated in these glazes, accompanied by a relatively high flux content, ranging from approximately 5.2 wt% to 15.79 wt%. This analysis reveals that the chemical composition of the glaze of high-temperature iron-series glazed wares from the Guangyuan kiln exhibits similarities to the composition of black glazed wares used throughout Chinese history [26], indicating its classification as a quintessential high-temperature iron-series glaze.

**Figure 2:**
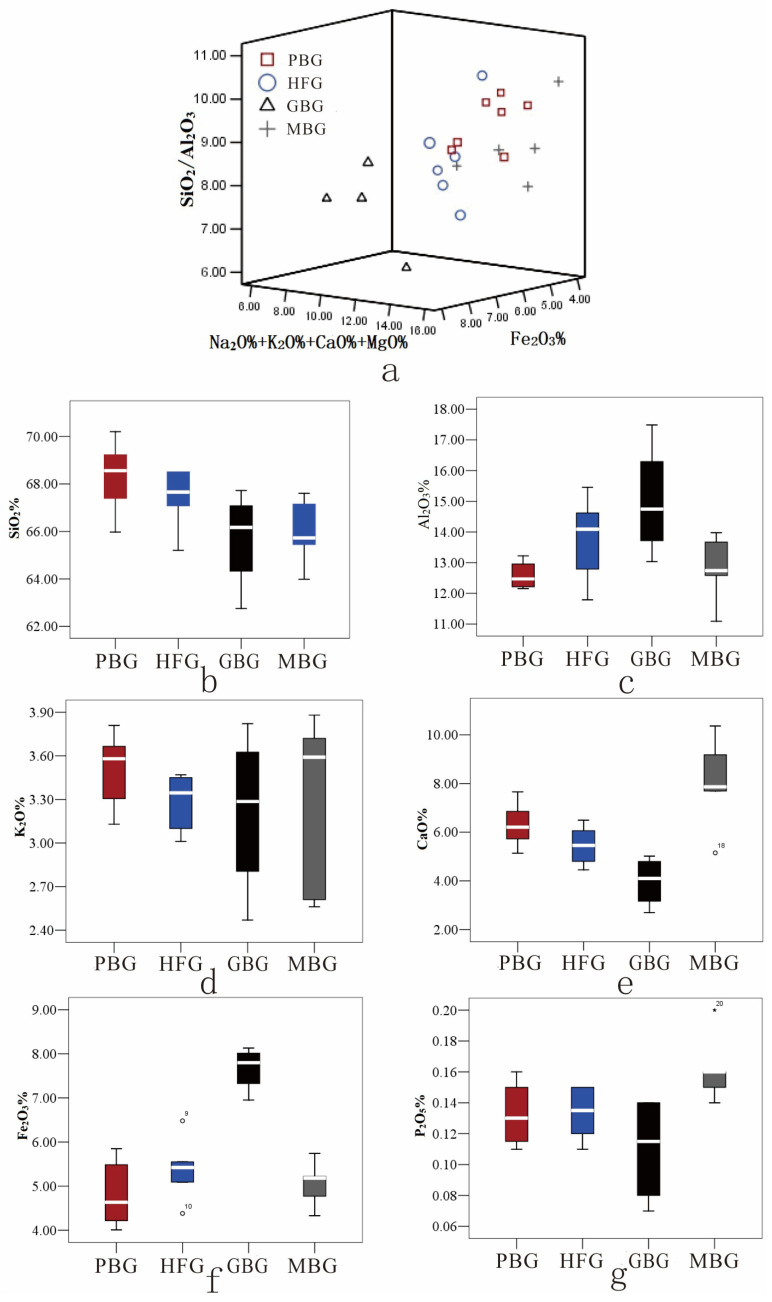
Scatter plot and box plot of the chemical composition of the Guangyuan kiln black glaze porcelain. (**a**) Scatter plot; (**b**) Box plot of SiO_2_ content; (**c**) Box plot of Al_2_O_3_ content; (**d**) Box plot of Fe_2_O_3_ content; (**e**) Box plot of P_2_O_5_ content, the ° in the diagram represents outliers; (**f**) Box plot of CaO content, the ° in the diagram represents outliers; (**g**) Box plot of K_2_O content. The * in the diagram represents outliers.

To delve into the discrepancies in glaze composition among pure black, hare’s fur, glossy brown, and matte brown glazed wares, SPSS v13.0 statistical software was employed to generate scatter plots and single-element box plots depicting the chemical composition of the four glaze types, as presented in Table S2 and Figure 2. The scatter plot in Figure 2 reveals that the chemical compositions of the four glaze types cluster within distinct regions. Specifically, the chemical compositions of the glaze of the pure black glazed ware are predominantly located in the high SiO_2_/Al_2_O_3_ and low Fe_2_O_3_ region; the chemical compositions of the glossy brown glaze occupy the low SiO_2_/Al_2_O_3_, high Fe_2_O_3_, and low flux region; that of the matte brown glaze are concentrated in the low iron and high flux region; while the hare’s fur glaze fall between them. The results of a *t*-test analysis comparing the major components of the glaze of glossy brown and matte brown glazed wares are presented in Table 3. Table 3 reveals significant differences between the two glaze types in their CaO, Fe_2_O_3_, and P_2_O_5_ content. Based on these findings, it can be further inferred that the glazes of the glossy and matte brown glazed wares utilized distinct glaze recipes. Moreover, the difference in surface coloration between the two glaze types is likely attributable to the variations in their Fe_2_O_3_ and CaO content.

Overall, the glaze of glossy brown glazed wares exhibits the highest Fe_2_O_3_ content, consistently exceeding 6.9 wt%, which is markedly higher than the Fe_2_O_3_ content observed in other ware glaze types. In contrast, pure black glazed wares possess the lowest Fe_2_O_3_ content, with an average of 4.84 wt%, while hare’s fur glazed and matte brown glazed wares fall between the two aforementioned entities. The elevated Fe_2_O_3_ content in glossy brown glazes provides an ample source of iron for crystal formation. The glaze of glossy brown glazed wares exhibits the lowest SiO_2_/Al_2_O_3_ ratio among the four glaze types, while pure black and matte brown glazed wares demonstrate higher ratios. Furthermore, matte brown glazes possess the highest flux content, averaging 14.62 wt%, with a CaO content averaging 8.05 wt%, considerably higher than that of other glaze types, where CaO content remains below 6.3 wt%. The higher fluxing agents and CaO content contribute to a reduction in glaze viscosity and facilitate the precipitation of anorthite crystals on the glaze surface [27]. The hare’s fur markings in the hare’s fur glaze are primarily characterized by yellow tones, indicating firing under oxidizing conditions [26]. Comparative analysis with Table S2 indicates that the chemical composition of the yellow hare’s fur markings and the black base glaze in the hare’s fur glaze are similar, except for a higher Fe_2_O_3_ content in the former. This suggests that the hare’s fur pattern is formed within a single firing of the same iron-based glaze, with the markings arising from the natural crystallization of iron oxide during the firing process, analogous to the production techniques employed at the Jian kilns for the hare’s fur glazed ware [1].

### 3.2. Glaze Microstructure

**Figure 3:**
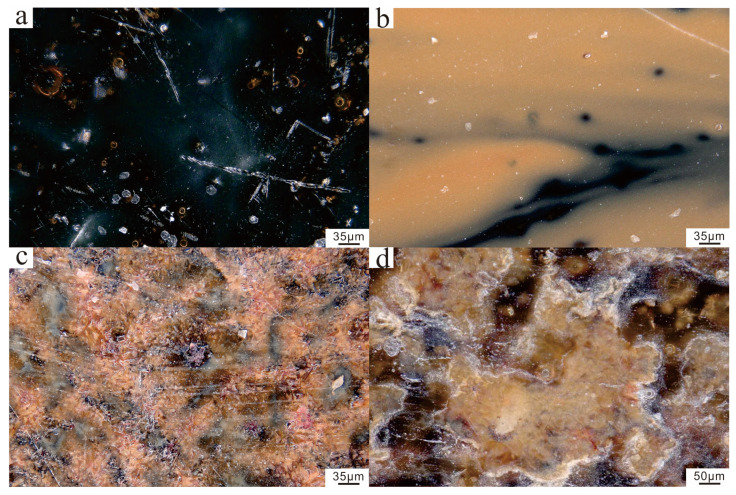
Optical microscope images. (**a**) Black glazed ware, (**b**) Hare’s fur glazed ware, (**c**) Glossy brown glazed ware, (**d**) Matte brown glazed ware.

Disparities in chemical composition result in distinct glaze surface microstructures between the black and brown glazed wares, leading to variations in glaze color and appearance. Representative samples of the pure black glazed ware (GY-7), hare’s fur glazed ware (GY-10), glossy brown glazed ware (GY-14), and matte brown glazed ware (GY-22) were meticulously selected. Figure 3 presents the ultra-depth-of-field microscopy images of the distinct types of samples, revealing notable distinctions between the glaze surfaces of the black and brown glazed wares. The glaze of pure black glazed wares boasts a sleek and highly lustrous surface of homogeneous black hue, punctuated by a scattering of yellow bubbles of varying sizes. The glaze of the hare’s fur glazed ware presents a flat surface adorned with yellow streaks devoid of significant crystal precipitation. In contrast, the glaze surface of brown glazed wares displays pronounced crystal formations. The glaze surface of glossy brown glazed wares features a predominantly reddish-brown surface with abundant yellowish-brown crystal precipitation and a relatively low glass phase content. The glaze surface of matte brown glazed wares possesses an uneven surface characterized by numerous light brown crystal clusters embedded within a dark brown matrix, along with a scattering of bubbles of various dimensions.

**Figure 4:**
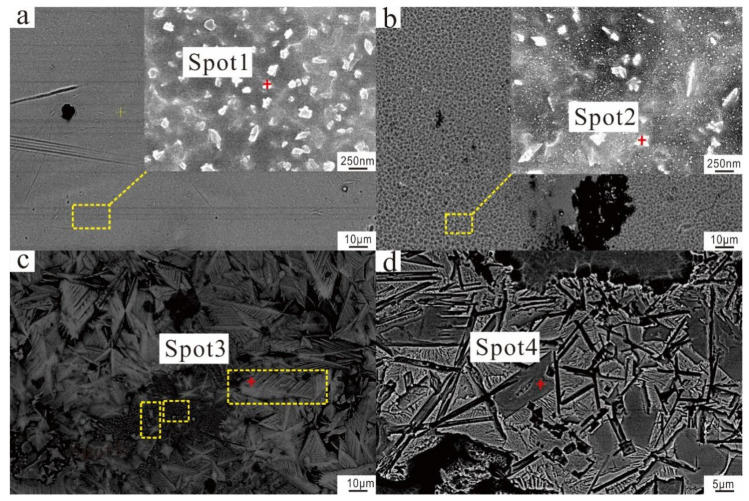
SEM images of crystal morphology on the surface of various high-temperature iron-series glazed wares (**a**) Pure black glazed ware, the image in the top right corner is the enlarged area of the yellow box, and the red dot indicates the test location for Spot 1 in Table 4. (**b**) Hare’s fur glazed ware, the image in the top right corner is the enlarged area of the yellow box, and the red dot indicates the test location for Spot 2 in Table 4. (**c**) Glossy brown glazed ware, there are three different types of crystals within the yellow frame, and the red dot indicates the test location for Spot 3 in Table 4. (**d**) Matte brown glazed ware, the red dot indicates the test location for Spot 4 in Table 4.

Figure 4 presents the SEM images of the surfaces of various high-temperature iron-series glazed wares, obtained using backscattered electron detection. The images reveal that both the glaze surfaces of pure black and hare’s fur glazed wares have precipitated a large number of irregular nanoscale grains. In the glaze surfaces of pure black glazed wares, the precipitated crystals are relatively small, ranging from 100 to 250 nm in size. Conversely, the hare’s fur glaze exhibits evidence of liquid-liquid phase separation within the streaked regions, characterized by a worm-like morphology. Crystallization is observed at the interface of these separated phases, yielding not only dendritic crystal flowers approximately 200–400 nm in size but also smaller crystal nuclei of around 30–40 nm. As Fe_2_O_3_ ascends with the bubbles to the glaze surface, it reaches supersaturation and precipitates. The high temperature and low viscosity of the glaze facilitate the flow of these precipitates, leading to the formation of hare’s fur streaks. Additionally, the yellow coloration of these streaks is attributed to the incomplete crystallization at the glaze surface, which lacks preferred orientation and results in the random growth of microcrystals [26]. These observations suggest that the formation mechanism of the hare’s fur glaze in the Guangyuan kiln aligns with the iron oxide crystallization induced by localized liquid-liquid phase separation at the glaze surface [1].

In contrast, both the glaze surfaces of glossy and matte brown glazed wares exhibit precipitation of significantly larger crystals. Specifically, the glaze surface of glossy brown glazed wares contains primarily yellowish-brown and micron-sized dendritic crystals, averaging around 25 µm in size. These crystals exhibit well-developed structures with parallel branches that grow in a periodic and orderly fashion, forming a two-dimensional planar arrangement. Their morphological characteristics bear a striking resemblance to the ε-Fe_2_O_3_ crystals observed in the oil spot glazes of the Song Dynasty Jian kiln, the brown glazed ware from Jiangxi Qilizhen, and the purple-gold glazed ceramic of the Qing Dynasty Jingdezhen kiln [28,29,30]. Furthermore, the oriented crystallization of the glaze yields a highly reflective and mirror-like surface, manifesting macroscopically as lustrous brown. Concurrently, a sparse distribution of smaller crystals was observed in the vicinity, and these crystals can be categorized into two distinct types based on their size and morphology. The first type comprises foliated crystals, with particle sizes ranging from a few micrometers to ten micrometers. The second type consists of smaller dendritic crystals, exhibiting approximately four to six branches, each around three to five micrometers in length. Wang et al.’s research [29] indicates that the growth process of dendritic ε-Fe_2_O_3_ crystals in the Southern Song dynasty brown glazed wares from Qilizhen begins with initial nucleation seeds developing into nanoscale spherical or irregularly shaped particles. These then evolve into two-dimensional dendritic structures with four, six, and eight primary branches, culminating in the formation of mature dendritic crystals. Based on the observed crystal types in the glaze surface of glossy brown glazed wares, it is hypothesized that the crystal growth process in this glaze is similar to that of the dendritic crystals found in the Southern Song dynasty brown glazed ware from Qilizhen. This process involves the initial formation of dendritic crystals approximately 3–5 µm in length, followed by the development of 4–6 branches, culminating in the growth of large-scale dendritic ε-Fe_2_O_3_ crystals. Further research will be conducted on this issue subsequently. Conversely, the light brown crystal clusters in the glaze of matte brown glazed ware predominantly consist of plate-like crystals with varying morphologies.

EDS analysis was conducted on the crystals observed on the surfaces of the four types of glazes, and the results are presented in Table 4. Elemental analysis via EDS indicates that the crystals present in all four glazes are iron-based. Among them, the glaze of the glossy brown glazed ware exhibited the highest Fe_2_O_3_ content within its crystalline structure, while that of the pure black glazed ware contained the least. The hare’s fur and matte brown glazed wares displayed intermediate Fe_2_O_3_ concentrations. Furthermore, a positive correlation was observed between crystal size and the Fe_2_O_3_ content within the glaze. Furthermore, significant quantities of Si and Al were detected, alongside trace amounts of Na, Mg, K, and Ca. These elements are likely derived from the vitreous matrix encompassing the crystals. It is noteworthy that the Ca content in matte brown glaze crystals is particularly high, reaching 14 wt%, while it does not exceed 8 wt% in other types of ceramic glaze crystals. This suggests that the crystals in the glaze of the matte brown glazed ware may contain not only iron-based crystals but also calcium-based crystals.

### 3.3. Iron Crystalline Phases and Crystallization Morphology

**Figure 5:**
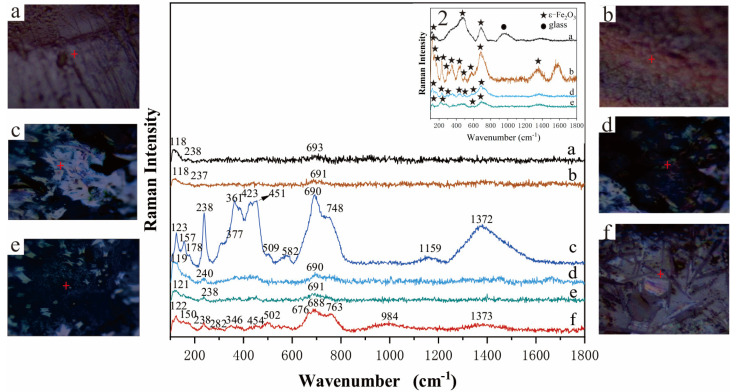
Raman spectra of dendritic crystals on the surface of black glaze and paste glaze samples from the Guangyuan kiln measured at low power. 2: Raman spectra of crystals tested at high power. (**a**) Granular crystals in pure black glaze; (**b**) Dendritic crystals in hare’s fur glaze; (**c**) Large dendritic crystals in glossy brown glaze; (**d**) Leaf−like crystals in glossy brown glaze; (**e**) Fine dendritic crystals in glossy brown glaze; (**f**) Crystals in matte brown glaze.

To further ascertain the crystal structures of the diverse morphologies observed on the glaze surfaces of black and brown glazed wares, Raman spectroscopy was employed to analyze the iron-bearing crystals. The results are presented in Figure 5. The glaze crystals of pure black and the crystalline flowers in the hare’s fur glaze and the leaf-shaped and fine branch-shaped crystals on the glossy brown glaze surface show similar and relatively weak Raman characteristic peaks, with relatively weak peaks only appearing at the bands near about 118, 238, and 690 cm^−1^. Raman spectroscopic analysis of the dendritic crystals found in the glaze surface of glossy brown glazed wares reveals distinct characteristic peaks, primarily at 123, 157, 178, 238, 361, 423, 451, 503, 582, 690, 748, 1159, and 1372 cm^−1^. A comparison with existing literature [28,29,30] indicates that these peaks are similar to those exhibited by the two-dimensional organized dendritic crystal structures found in the Song Dynasty JianKiln oil spot and hare’s fur glazed ware, Jiangxi Qilizhen brown glazed ware, and Qing Dynasty Jingdezhen Imperial Kiln Factory purple-gold glazed ceramic. This further corroborates that the micron-sized dendritic crystals within the glossy brown glaze are indeed ε-Fe_2_O_3_ crystals [31,32]. In order to clarify the types of grains on the surfaces of pure black glaze, the crystalline flowers in the hare’s fur glaze and the leaf-shaped and fine branch-shaped crystals on the glossy brown glaze surface, the samples were re-tested by Raman spectroscopy. The results with increased Raman test power are shown in Figure 5 (2). It was found that the Raman spectra of these crystals showed strong characteristic peaks, which displayed the same characteristics as the characteristic peaks of ε-Fe_2_O_3_. This further indicates that the crystals on the glaze surfaces of pure black glaze, hare’s fur glaze, and shiny sauce glaze are all ε-Fe_2_O_3_ crystals. Studies have shown that larger ε-Fe_2_O_3_ crystal sizes in brown glazed ware glazes contribute to a reddish hue [30]. The glaze surfaces of glossy brown glazed wares, predominantly composed of well-crystallized and micron-sized ε-Fe_2_O_3_ crystals, exhibit a lustrous brownish-red appearance.

Through microscopic examination and Raman spectroscopic analysis, we observed that the glaze surface of glossy brown glazed wares is predominantly composed of well-developed and micron-sized dendritic crystals, exhibiting pronounced Raman peaks. Conversely, underdeveloped crystals displayed significantly weaker Raman peaks. The glazes of pure black and hare’s fur glazed wares are primarily composed of underdeveloped nanocrystalline grains, resulting in weak Raman peaks.

The light brown crystalline spectrum of matte brown glazed wares also displays strong characteristic peaks. However, it differs from the Raman spectra of the aforementioned samples. Raman peaks situated at 150, 282, 502, 563, 676, 763, and 984 cm^−1^ are closely aligned with the characteristic peaks of anorthite [33], suggesting its presence within the glaze. Furthermore, peaks at 122, 238, 346, 454, 688, and 1373 cm^−1^ exhibit proximity to the characteristic peaks of ε-Fe_2_O_3_, indicating the presence of ε-Fe_2_O_3_ in this region as well. This is because the high CaO content in the glaze of the matte brown glazed ware provides an abundant calcium source for the formation of anorthite. During the high-temperature firing and cooling process, the glaze surface facilitates the precipitation of a significant amount of anorthite crystals. These crystals obscure the transparent texture of the glassy phase on the glaze surface, resulting in an opacified or milky appearance. Furthermore, the presence of ε-Fe_2_O_3_ crystals on the glaze surface contributes to its matte brown color.

**Figure 6:**
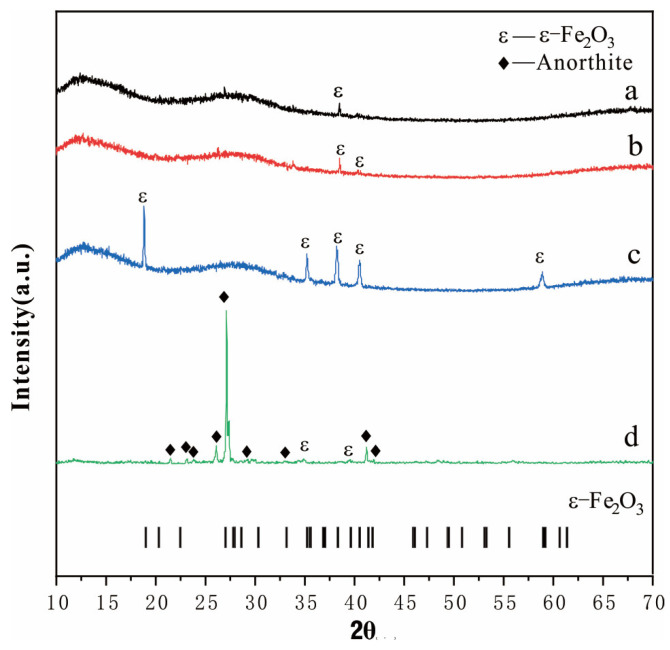
XRD patterns of the surface of black and brown glazed ware samples from the Guangyuan kiln. (**a**) Pure black glazed ware; (**b**) Hare’s fur glazed ware; (**c**) Glossy brown glazed ware; (**d**) Matte brown glazed ware.

To further identify the crystalline phases present on the glaze surface of black-glazed and brown-glazed wares, XRD analysis was performed on the crystals, as illustrated in Figure 6. The diffractograms reveal that both the crystals of the pure black and the hare’s fur glazed ware are primarily composed of an amorphous glassy phase, exhibiting indistinct diffraction peaks. Notably, the crystals of the pure black glazed ware display only a weak diffraction peak corresponding to ε-Fe_2_O_3_ at 38.5°. Those of the hare’s fur glaze and brown glazed wares exhibit weak ε-Fe_2_O_3_ diffraction peaks at 38.5°and 40.4° [34], suggesting a limited presence of crystalline phases with underdeveloped crystal structures in both glazes. The crystals of the brown glazed ware exhibit pronounced diffraction peaks, indicative of a higher degree of crystallinity. The XRD pattern of the glaze surface of glossy brown glazed wares reveals diffraction peaks at 18.9°, 35.3°, 38.3°, 40.5°, and 59°, all corresponding to the ε-Fe_2_O_3_ phase [33,34]. These peaks are sharp and intense, further confirming the high crystallinity of ε-Fe_2_O_3_ on the glaze surface of the glossy brown glazed ware, which aligns with the observation of abundant large-sized dendritic crystals through SEM observation. Furthermore, the glaze surface of matte brown glazed wares exhibits the presence of two distinct crystalline phases. In addition to the characteristic peaks corresponding to anorthite [29], two faint diffraction peaks indicative of ε-Fe_2_O_3_ are also observed. This confirms that the crystalline phase in the matte brown glaze is predominantly anorthite, with a minor presence of ε-Fe_2_O_3_ crystals.

Based on the compositional, structural, and phase analyses, it can be concluded that the glaze of pure black glazed wares has the lowest Fe_2_O_3_ content averaging 4.69 wt%, and its surface exhibits the precipitation of nano-sized iron-bearing crystals. The glaze of hare’s fur glazed wares exhibits a slightly higher Fe_2_O_3_ content than the pure black glazed ware, averaging 5.44 wt%, and precipitates slightly larger crystals. However, due to incomplete crystal development, both their Raman spectra and diffraction peaks are less pronounced. The high Fe_2_O_3_ content in the glossy brown glaze, averaging 7.67 wt%, fostered a supersaturated concentration, promoting the precipitation and growth of iron-based crystals. Consequently, the glaze surface exhibited a proliferation of well-developed, micron-sized, and dendriticε-Fe_2_O_3_ crystals, manifesting a macroscopically observable reddish-brown luster. Additionally, the glaze contained leaf-like and delicate dendritic crystal formations. The CaO content in the glaze of matte brown glazed wares is notably higher than in other wares, averaging 8.78 wt%. During the high-temperature firing and subsequent cooling process, a significant quantity of anorthite crystals develops, imparting a distinctive opaque to the glaze surface. Furthermore, the presence of Fe_2_O_3_ in the glaze leads to the precipitation of ε-Fe_2_O_3_ crystals, imbuing the surface with a brown hue. These findings strongly support the correlation between the Fe_2_O_3_ and CaO content in both the glossy and matte-brown glazed wares from the Guangyuan kiln and their respective color characteristics.

## 4. Conclusions

(1) The bodies of high-temperature iron-series glazed wares produced in the Guangyuan kiln were crafted using locally sourced and iron-rich clay minerals. The average iron content in these bodies, at 3.59 wt%, is lower than that found in the Jian kiln wares, resulting in a predominantly greyish-white hue for the Guangyuan kiln ware bodies. Notably, the raw materials used for the ceramic bodies remained consistent across the pure black, hare’s fur, glossy brown, and matte brown glazed wares.
(2) The Fe_2_O_3_ content varied among the four types of Guangyuan kiln wares. The glaze of glossy brown glazed wares exhibited the highest average Fe_2_O_3_ content at 7.67 wt%, while pure black glazed wares had the lowest at 4.84 wt%. Hare’s fur and matte brown glazed wares fell among them. This high Fe_2_O_3_ content provides an abundant source of iron for glaze crystallization, promoting the formation of iron-bearing crystals on the glaze surface. Notably, the glaze of matte brown glazed wares displayed the highest levels of both CaO and total fluxing agents.
(3) The glaze surfaces of the four types of Guangyuan kiln wares exhibit precipitation of ε-Fe_2_O_3_ crystals. The glaze surface of pure black glazed wares displayed abundant nano-sized ε-Fe_2_O_3_ grains, while hare’s fur glazed wares featured a combination of dendritic crystal flowers and small nuclei on the surface of the “hare’s fur” markings. The glaze surface of the glossy brown glazed ware exhibited large, two-dimensional, and dendritic crystals, whereas the matte brown glazed ware primarily featured calcium feldspar crystals with a minor presence of ε-Fe_2_O_3_ crystals. As the Fe_2_O_3_ content increased, the size of the ε-Fe_2_O_3_ crystals increased, accompanied by a corresponding intensification of Raman peak intensity and sharper diffraction peaks. These observations indicate that a higher Fe_2_O_3_ content promotes superior ε-Fe_2_O_3_ crystal development and crystallinity within the glaze.

## Figures and Tables

**Table 1:** Macroscopic characteristics of the samples.

Type	Description
Pure black glazed ware (PBG)	A black glaze covers the surface, with a slip applied between the glaze and body on some samples. The gray body exhibits a relatively dense composition.
Hare’s fur glazed ware (HFG)	Both the interior and exterior surfaces are coated in a black glaze, adorned with yellow hare’s fur markings. The body is gray and exhibits a dense composition.
Glossy brown glazed ware (GBG)	The interior and exterior are enveloped in a lustrous, reddish-brown glaze. The body, composed of gray clay, boasts a dense and robust structure.
Matte brown glazed ware (MBG)	The interior and exterior are coated in a brown glaze, though the application ceases shy of the base. The body, comprised of gray clay, displays a dense and robust composition.

**Table 2:** Mean chemical composition and consistency test of the bodies of black and brown glazed wares.

Type	Na_2_O	MgO	Al_2_O_3_	SiO_2_	K_2_O	CaO	TiO_2_	Fe_2_O_3_
Black glazed ware	0.52	1.29	22.05	67.45	2.49	0.59	0.86	3.73
Brown glazed ware	0.52	1.09	25.29	64.65	2.20	0.84	1.02	3.38
T	0.00	1.08	−1.40	1.36	0.96	−0.98	−0.89	0.53
P	1.00	0.29	0.17	0.18	0.34	0.33	0.38	0.60

Note: The chemical composition of the oxides in black glazed porcelain and brown glazed porcelain is given as the mean, with units in wt%. *p* < 0.05 indicates a significant difference.

**Table 3:** Mean Values and Consistency Tests of the Chemical Compositions of Matte and Glossy Brown Glazes from the Guangyuan Kiln.

Type	Quantity	Na_2_O	MgO	Al_2_O_3_	SiO_2_	K_2_O	CaO	TiO_2_	Fe_2_O_3_	MnO	P_2_O_5_
Matte brown glazed ware	5	0.52	1.29	22.05	67.45	2.49	0.59	0.86	3.73	0.14	0.16
Glossy brown glazed ware	4	0.52	1.09	25.29	64.65	2.20	0.84	1.02	3.38	0.12	0.11
T	-	0.00	1.08	−1.40	1.36	0.96	−0.98	−0.89	0.53	0.85	2.67
P	-	1.00	0.29	0.17	0.18	0.34	0.33	0.38	0.60	0.42	0.03

**Table 4:** EDS Elemental Composition of Surface Crystals on the Guangyuan Kiln high-temperature iron glazed ware.

No.	Na	Mg	Al	Si	P	K	Ca	Ti	Mn	Fe	Sum
Spot1	1.4	1.8	11.1	60.9	1.6	5.7	8	1	-	8.6	100.1
Spot2	1.84	2.17	10.03	41.81	-	3.51	4.35	0.50	0.33	35.45	99.99
Spot3	-	1.07	10.55	41.59	-	3.36	2.91	0.92	-	39.60	100
Spot4	1.2	2	10.5	51.1	1.7	5.6	14	1	-	12.9	100

## Data Availability

The original contributions presented in the study are included in the article and Supplementary Material, further inquiries can be directed to the corresponding author.

## Supplementary Materials

The following supporting information can be downloaded at: https://www.mdpi.com/article/10.3390/ma17246221/s1, Table S1: Chemical composition of the body with iron-series glazed ware from Guangyuan kiln; Table S2: Chemical composition of the glaze with iron-series glazed ware from Guangyuan kiln.
